# Editorial: Mild cognitive impairment and cognitive aging

**DOI:** 10.3389/fnbeh.2026.1862932

**Published:** 2026-06-04

**Authors:** Eleni Konsolaki, Chrysanthi Nega, Ion Beratis, Miriam A. Hickey, Panagiotis Kourtesis

**Affiliations:** 1 Department of Psychology, Deree-The American College of Greece, Athens, Greece; 2 Faculty of Medicine, Institute of Biomedicine and Translational Medicine, University of Tartu, Tartu, Estonia; 3 Department of Psychology, National and Kapodistrian University of Athens, Athens, Greece; 4 Department of Psychology, The University of Edinburgh, Edinburgh, United Kingdom

**Keywords:** biomarkers, cardiometabolic health, cognitive aging, mild cognitive impairment, physical activity, prevention, risk factors, screening

Mild cognitive impairment (MCI) refers to a measurable decline in one or more cognitive abilities (such as memory, attention, or executive function) that exceeds what would be expected with normal aging but does not substantially impair independent daily activities. It is regarded as a transitional state between normal aging and dementia, with an elevated risk of progression over time. In geriatric population the prevalence was found 23.7% (Salari et al., 2025). Studying MCI is important not only because it may represent an early stage of a neurodegenerative disease, but also because it is a heterogeneous and evolving condition influenced by a range of biological, behavioral, and clinical factors. Accordingly, contemporary MCI research is moving beyond narrow diagnostic questions toward broader models that can better explain vulnerability, improve early identification, and inform care (Bonvino et al., 2025). Therefore, the key challenge is not only to identify MCI, but also to understand its varied determinants and integrate this knowledge into clinically meaningful approaches for assessment and management. This Research Topic addresses that challenge by assembling 12 papers that collectively position MCI as a multidimensional phenomenon. Instead of viewing cognition as an isolated endpoint, these studies situate MCI within broader epidemiological, systemic, behavioral, neurobiological, and translational frameworks.

Four interconnected domains provide a framework through which these contributions can be understood: (i) population burden and cardiometabolic risk architecture; (ii) lifestyle, symptoms, and comorbidity-linked vulnerability; (iii) biomarkers and practical early detection tools; and, finally, (iv) clinical stratification and intervention-oriented translation. This framework is valuable because it highlights that MCI research is no longer limited to describing cognitive deficits in isolation. Instead, the field is shifting toward models that situate cognitive impairment within broader health trajectories and evaluate it through multiple, converging sources of evidence. The studies in this Research Topic approach the challenge from multiple angles, and their strength lies in showing how insights from different levels of analysis can inform and complement one another.

An initial set of papers delineates the scope of the issue and places MCI within the wider framework of cardiometabolic risk. Collecting data from Chinese older adults, Wu et al. provide a prevalence-oriented anchor for the topic by documenting that cognitive impairment is highly prevalent among Chinese adults aged ≥60 years (44.0%) and is strongly associated with demographic, socioeconomic, and lifestyle factors such as age, education, residence, and social engagement. This epidemiological perspective is complemented by Zhou et al., who link the atherogenic index of plasma with cognitive impairment in middle-aged and older adults, drawing attention to the contribution of metabolic and vascular processes to age related cognitive decline and by Chen et al., who examine the impact of visceral adiposity index on cognitive impairment and cognitive trajectories. These studies go beyond simply linking metabolic markers to cognition; they indicate that cognitive aging cannot be fully understood without considering the broader physiological context in which decline occurs. Together, this body of work underscores both the significance of the problem and the need to position cardiometabolic health as a central, rather than peripheral, component of MCI research.

A second cluster brings the discussion closer to daily life, routine care, and modifiable vulnerability. Zhang et al. examine physical activity levels among Chinese older adults and provide one of the clearest prevention-focused contributions in the Research Topic by linking an actionable behavioral factor to MCI. Gan et al. show that poorer sleep quality is associated with MCI in patients with chronic heart failure, a population in whom cognitive decline is likely to be shaped by substantial physiological burden and clinical complexity. Liu X. et al. further broaden the field by showing that constipation symptoms are associated with worse cognitive outcomes in older adults without dementia. The significance of these papers lies in extending MCI research into aspects of everyday life that are directly relevant to routine clinical practice. Factors such as physical activity, sleep quality, and gastrointestinal symptoms are not merely incidental findings but represent modifiable and clinically accessible correlates that can enhance early risk assessment and support more prevention-focused approaches.

A third group of articles addresses the challenge of achieving earlier detection. Liu Y. et al. synthesize 25 studies demonstrating that olfactory testing has moderate to high diagnostic utility for both Alzheimer's disease and mild cognitive impairment, supporting its value as a simple, non-invasive, and cost-effective screening approach. Hussen et al. demonstrate that a nomogram integrating non-invasive retinal and visual biomarkers can achieve high accuracy in the early detection of MCI, highlighting the potential of ocular measures as practical screening tools in clinical settings. Yang et al. review the potential of functional near-infrared spectroscopy as a promising tool for identifying MCI in older adults with further standardized, large-scale studies required to establish its diagnostic utility. Taken together, these studies illustrate a field actively exploring more accessible and scalable approaches to screening. They also underscore that no single marker is likely to suffice, pointing instead to a future in which early detection relies on integrated combinations of sensory, ocular, physiological, and neuroimaging indicators that enhance sensitivity while remaining feasible for both research and clinical use.

The final cluster points toward stratification and translation. Qi et al. demonstrate that patients with MCI, stratified by cerebrospinal fluid biomarkers, exhibit distinct patterns of spontaneous brain activity, underscoring the biological heterogeneity that may be concealed within the broad diagnostic category of MCI. Pistorio et al. add a clinically relevant management perspective by demonstrating that higher anticholinergic drug use was associated with poorer cognitive performance and more severe psychiatric symptoms, suggesting that reducing anticholinergic burden may alleviate psychiatric symptoms in cognitive disorders. In parallel, Gambella et al. present the Silver Agri Age project, a Montessori-inspired social intervention protocol for older adults with MCI, thereby shifting attention toward implementation and real-world intervention design. This is a particularly important concluding set, as it highlights the direction the field needs to take, toward subgroup-sensitive models, clinically meaningful management factors, and interventions that extend beyond description to real-world impact. In this way, the Research Topic not only characterizes MCI but also begins to define the conditions under which more personalized approaches to care may be achieved.

The main contribution of this Research Topic lies in its integration across multiple levels of analysis. MCI is presented not as a uniform or purely brain-based condition, but as a multidimensional condition shaped by the interaction of population-level burden, cardiometabolic risk, everyday behaviors, somatic symptoms, accessible biomarkers, and clinically meaningful heterogeneity. This broader perspective challenges the notion that progress in MCI research will come from a single defining marker or explanatory model, suggesting instead that advances are more likely to emerge from integrative approaches linking epidemiology, physiology, behavior, and neurobiology. Such an approach is also more aligned with clinical reality, where patients present not as isolated biomarkers or symptoms but as complex phenotypes modulated by risk, impairment, overall health, and lived experience. In this sense, the Research Topic underscores the necessity of research methodologies that better capture this multidimensional clinical reality.

Future work should build directly on the directions opened by these contributions. Longitudinal designs will be essential for clarifying temporal and potentially causal pathways linking systemic health, behavior, and cognitive outcomes (Ding et al., 2023). Multimodal studies are needed to test how population risk indicators, modifiable lifestyle factors, and biomarker-based tools can be combined into more robust models of early identification. Equally important will be the development of subgroup-sensitive frameworks capable of distinguishing clinically meaningful forms of MCI rather than treating the category as homogeneous (Machulda et al., 2019). Finally, intervention work must continue to expand, particularly where it integrates biological insight with practical feasibility and real-world applicability. Collectively, the papers in this Research Topic move the field toward a more comprehensive and clinically useful understanding of mild cognitive impairment in aging. Their shared message is that progress in MCI research will depend on integration across methods, levels of analysis, and translational priorities.
